# Correction: Collateral impacts of pandemic COVID-19 drive the nosocomial spread of antibiotic resistance: A modelling study

**DOI:** 10.1371/journal.pmed.1004268

**Published:** 2023-07-19

**Authors:** The PLOS Medicine Staff

There is an error in the shading of Table 1. Please see the correct Table 1 below. The publisher apologizes for the error.

**Table 1:** Responses to COVID-19 included in the transmission model. See Methods for description of how COVID-19 response parameters are implemented in model equations. Rows describing policy responses are shaded blue, and rows describing caseload responses are shaded grey. Symptomatic refers to COVID-19 symptoms among individuals infected with SARS-CoV-2. COVID-19; Coronavirus Disease 2019; HCW, healthcare worker; IPC, infection prevention and control; PPE, personal protective equipment; SARS-CoV-2, Severe Acute Respiratory Syndrome Coronavirus 2.

COVID-19 response		Evidence	Model implementation	Category/Cause	Interpretation	
					τ = 0	τ = 1
τ_*as*_	Abandoned stewardship	Reduction in antibiotic stewardship activities [17]	Increased proportion of patients exposed to antibiotics (*A*)	Antibiotics/Caseload	No change in antibiotic use	Large increase in antibiotic use during COVID-19 surges
τ_*cp*_	COVID-19 prescribing	COVID-19 patients receive high rates of antibiotic prescription [18]	Increased proportion of symptomatic COVID-19 patients exposed to antibiotics (*A*_*I*_)	Antibiotics/Policy	No excess antibiotic prescribing among symptomatic patients	All symptomatic patients receive antibiotics
τ_*cd*_	Care disorganization	Compromised ability of HCWs to adhere to IPC best practices (e.g., due to increased workload, PPE shortages) [14]	Increased daily rate of at-risk patient–HCW contact (*κ*^*pat→hcw*^)	Contact/Caseload	No change in contact behaviour	Large increase in at-risk patient–HCW contact during COVID-19 surges
τ_*pl*_	Patient lockdown	Social interactions among patients limited or forbidden [19]	Decreased daily rate of patient–patient contact (*κ*^*pat→pat*^)	Contact/Policy	No change in contact behaviour	Elimination of all patient–patient contact
τ_*um*_	Universal masking	HCWs and patients wear face masks to prevent transmission [20]	Decreased SARS-CoV-2 transmissibility per contact (*π*_*V*_)	IPC/Policy	No change in SARS-CoV-2 transmissibility	SARS-CoV-2 rendered nontransmissible (perfect mask effectiveness)
τ_*hh*_	Hand hygiene	Increase in HCW handwashing performance [21]	Increased hand hygiene compliance (*H*)	IPC/Policy	No change in hand hygiene compliance	Perfect hand hygiene compliance
τ_*cs*_	COVID-19 stays	COVID-19 patients remain in healthcare facility until recovered [22]	Decreased discharge rate for symptomatic COVID-19 patients (*μ*_*I*_)	Disease/Caseload	No impact of SARS-CoV-2 infection on patient length of stay	All patients remain in hospital while symptomatic
τ_*ss*_	Staff sick leave	HCWs with COVID-19 stay home from work [23]	A proportion of symptomatic HCWs removed from population for 7 days (until recovered)	Disease/Caseload	No symptomatic staff go on sick leave	All symptomatic staff go on sick leave after being infectious for 1 day
τ_*ra*_	Reduced admission	Decreased number of hospital admissions during COVID-19 surges [24]	Decreased patient admission rate (*μ*)	Admission/Caseload	No change in patient admissions	Large reduction in patient admissions during COVID-19 surges
τ_*sc*_	Sicker casemix	Elective admissions delayed or cancelled during COVID-19 surges, restricting admissions to more critically ill patients [10]	Increased rate of antibiotic-resistant bacterial carriage among patient admissions ($f_{C^{R}}$)	Admission/Caseload	No change in the probability of colonization upon admission	Large increase in the probability of colonization with resistant bacteria upon admission during COVID-19 surges
