# Supplementary material for: Collateral impacts of pandemic COVID-19 drive the nosocomial spread of antibiotic resistance: A modelling study
Source: PLoS Med. 2023 Jun 5;20(6):e1004240. doi: 10.1371/journal.pmed.1004240 (PMC10241372; doi:10.1371/journal.pmed.1004240)
Supplement: Attachment — Submitted filename: smith.pdf [file pmed.1004240.s002.pdf]

This paper uses a mathematical model to describe the relationship between response to COVID–19 and nosocomial spread of antibiotic resistance.

I was asked for a statistical report and I interpret that to include all aspects of the design and conduct of the study.

Points of detail

Page 5 Assuming no interaction between infection and colonisation seems a strong assumption unless there is evidence to support it.

Page 6 Is death included here as a subset of discharge? Perhaps clarify one way or the other.

Page 9 Good to see the package being properly cited (and also in the supplementary material) but not in the caption to Figure 3 on page 13.

Page 9 The model was populated using data from a long–term hospital but later the model is applied to other quite different settings. I think the validity of this needs to be discussed further. How typical is a long–term hospital of health care settings in general?

Page 15, Figure 4b I do not see how the diamond representing "all" can be positioned on the x–axis almost identically to the square for "policy" when there are clearly many points and the nabla for "caseload" well to the right of either. Is this due to excessive overprinting or have I misunderstood what the graph is supposed to be telling me?

Discussion The authors motivate their work by pointing to conflicting results from empirical analysis of various datasets. Do their simulations go any way to resolving the different findings?

Code This is the best structured and documented set of code I have seen submitted as supplementary material.

I am not an expert in infectious disease control so I am unable to comment on whether the range of values which the authors have used to populate their model are plausible ones or whether the specific linkages are appropriate. It would be important to have a view from the clinical reviewers on this. Given the degree of detail here and the transparent code presented then anyone who subsequently disagrees with the authors' decisions can always re–run the model with new values.

Michael Dewey
