# Supplementary material for: Collateral impacts of pandemic COVID-19 drive the nosocomial spread of antibiotic resistance: A modelling study
Source: PLoS Med. 2023 Jun 5;20(6):e1004240. doi: 10.1371/journal.pmed.1004240 (PMC10241372; doi:10.1371/journal.pmed.1004240)
Supplement: Attachment — Submitted filename: retour_aux_reviewers.docx [file pmed.1004240.s003.docx]

We sincerely thank the combined efforts of the reviewers and editorial team, who have raised excellent points and helped us to improve our article.

**GENERAL**
Please respond to all editor and reviewer comments detailed below, in full

Please find our responses below, with changes to the manuscript highlighted in yellow.

Please remove the statements detailed at lines 26, 28 and 30 and include this information only in the manuscript submission form.

These statements have been deleted.

**ABSTRACT**
Abstract Background: Please ensure that the final sentence clearly states the study question.

We propose a revised sentence stating the study question (line 36):

We sought to evaluate how such collateral impacts of COVID-19 impacted the nosocomial spread of MRB in an early pandemic context.

**Abstract Methods and Findings:** as written precise details are rather vague and the text reads more like an introduction rather than a clear description of what you did and what you found.  We appreciate that is difficult to describe full details of the models/simulations concisely but we suggest some revision of this section in mind of the below points.

The abstract has been substantially rewritten according to the provided guidance.

Please use past tense i.e. were not are, for example - line 43 suggest “We conducted a mathematical modelling study to investigate…” or something similar, and line 48 suggest “…model simulations were conducted…”

The past tense is now used throughout the abstract.

It may be helpful to include come more specific details, if possible, of (at least some) model parameters, the different hospital settings simulated, varying ratios of patients and healthcare staff, proportions with COVID etc such that the reader gains some contextual insights

More specific details regarding model parameters are now provided (from line 41):

Responses to COVID-19 were captured mechanistically via a range of parameters that reflect impacts of SARS-CoV-2 outbreaks on factors relevant for pathogen transmission. COVID-19 responses include both “policy responses” willingly enacted to limit SARS-CoV-2 transmission (e.g. universal masking, patient lockdown, reinforced hand hygiene) and “caseload responses” unwillingly resulting from surges in COVID-19 caseloads (e.g. abandonment of antibiotic stewardship, disorganization of infection control programmes, extended length of stay for COVID-19 patients).

How did you decide which “diverse bacterial species” to investigate/simulate, can you give some examples of the major players and justifications perhaps

The conceptualization of “diverse bacterial species” and “diverse healthcare settings” has been clarified (from line 51):

The first set of simulations represents diverse MRB and nosocomial environments, accounting for high levels of heterogeneity across bacterial parameters (e.g. rates of transmission, antibiotic sensitivity and colonization prevalence among newly admitted patients) and hospital parameters (e.g. rates of inter-individual contact, antibiotic exposure and patient admission/discharge).

Please clearly define the main outcome measures for the reader

The main outcome measures are now defined (from line 47):

We conducted two main sets of model simulations, in which we quantified impacts of SARS-CoV-2 outbreaks on MRB colonization incidence and antibiotic resistance rates (the share of colonization due to antibiotic-resistant versus -sensitive strains).

PLOS Medicine requires that the main results are quantified with 95% CIs and p values.  We understand the use of 95% UIs in a study of this design – please include these when reporting outcomes. Please see below also.

The main results are now quantified as proposed (from line 55):

On average, COVID-19 control policies coincided with MRB prevention, including 28.2% [95% uncertainty interval: 2.5%, 60.2%] fewer incident cases of patient MRB colonization. Conversely, surges in COVID-19 caseloads favoured MRB transmission, resulting in a 13.8% [-3.5%, 77.0%] increase in colonization incidence and a 10.4% [0.2%, 46.9%] increase in antibiotic resistance rates in the absence of concomitant COVID-19 control policies. When COVID-19 policy responses and caseload responses were combined, MRB colonization incidence decreased by 24.2% [-7.8%, 59.3%] while resistance rates increased by 2.9% [-5.4%, 23.2%].

**STATISTICAL REPORTING**
We note the reporting of negative values but also the use of hyphens to separate upper and lower bounds.  Suggest the use of commas instead to avoid any confusion between hyphens and negative values.

We have adopted this notation throughout.

As above, PLOS Medicine requires that the main results are quantified with 95% CIs and p values, including in the abstract.  We understand and accept the use of 95% UIs in a study of this design.  When reporting p values please report as p<0.001 or where higher, as p=0.002, for example.  If not reporting p values, for the purpose of transparent data reporting please clearly state the reasons why not. 

We now include the following statement in the Methods explaining why p-values are not considered (from line 326):

P-values are not reported due to lack of interpretability for simulated data.

**AUTHOR SUMMARY**
At this stage, we ask that you include a short, non-technical Author Summary of your research to make findings accessible to a wide audience that includes both scientists and non-scientists. The Author Summary should immediately follow the Abstract in your revised manuscript. This text is subject to editorial change and should be distinct from the scientific abstract.
It may help you to review some examples from published papers on our website here:
<https://journals.plos.org/plosmedicine/>
Please see our author guidelines for more information: <https://journals.plos.org/plosmedicine/s/revising-your-manuscript#loc-author-summary>

An author summary has been provided.

**INTRODUCTION**
Line 100: “Findings demonstrate….” Please either remove or move this sentence to the discussion section of the manuscript, as it reports the study outcomes.

This sentence has been removed.

**METHODS and RESULTS**
We ask that the following details [derived from Geoffrey P Garnett, Simon Cousens, Timothy B Hallett, Richard Steketee, Neff Walker. Mathematical models in the evaluation of health programmes. (2011) Lancet DOI:10.1016/S0140-6736(10)61505-X.] be included with all modelling studies.  We think that that you have included all relevant information but please review the list below and amend where necessary:

All elements are already provided.

**FIGURES**
Please consider avoiding the use of green and/or red to ensure that your figures are accessible to those with color blindness

The colour scale for Figure 4A (and associated supplementary plots) has been updated using a scale optimized for colour blindness (https://www.nature.com/articles/nmeth.1618).

Please ensure that all abbreviations are defined in an appropriate caption for example HCW in figure 2

HCW has been defined in Figure 2’s caption.

Figure 3: in the caption you detail confidence interval but in the text you detail uncertainty interval , please clarify/revise accordingly

Boxplot notches provide a visual approximation of the 95% confidence interval of a distribution median. This is distinct from the 95% uncertainty intervals used to report outcome distributions from our simulations. To avoid confusion, we have removed the statement about visual interpretation of boxplot notches from all boxplot figure captions.

**REFERENCES**
Please see our website for reference guidelines:
<https://journals.plos.org/plosmedicine/s/submission-guidelines#loc-references>
Please ensure that for in-text reference callouts, citations are placed in square brackets preceding punctuation, for example line 72 should read “…including sexually transmitted infections (e.g. HIV) [1], vector-borne illnesses…” please check and amend throughout where relevant.

These have been amended throughout.

Please ensure that the bibliography uses journal name abbreviations found in the National Center for Biotechnology Information (NCBI) databases. Please ensure that up to but no more than 6 author names are listed followed by et al, in the event that more than 6 authors contribute to a study. Please amend where necessary including the supporting files

The main manuscript bibliography did follow these conventions, but not the supplementary bibliography, which has been updated accordingly.

**Comments from the reviewers:**

**Reviewer #1: Michael Dewey** This paper uses a mathematical model to describe the relationship between response to COVID-19 and nosocomial spread of antibiotic resistance. I was asked for a statistical report and I interpret that to include all aspects of the design and conduct of the study.

We thank the reviewer for his time and careful review of our work.

Points of detail

**Page 5** Assuming no interaction between infection and colonisation seems a strong assumption unless there is evidence to support it.

Indeed, important within-host interactions between certain respiratory viruses and respiratory bacteria have been reported (e.g. impacts of influenza infection on invasive disease due to *Streptococcus pneumoniae*). However, in the case of SARS-Cov-2, there is as yet relatively little evidence to support a biological interaction between SARS-CoV-2 infection and bacterial colonization at the host level. For instance, in meta-analyses, 8.6% of COVID-19 patients experienced bacterial coinfection [Langford et al., *Clin Microbiol Infect* 2021] compared to 23% of influenza patients [Klein et al*., Influenza Other Respir Viruses* 2016]. However, we do agree that this is nonetheless an important assumption, motivating the following comment in our discussion (from line 610):

For simplicity, and due to limited evidence of a strong association between SARS-CoV-2 and bacterial coinfection [[14]](https://sciwheel.com/work/citation?ids=10265615&pre=&suf=&sa=0&dbf=0), our model assumes no impact of SARS-CoV-2 infection on bacterial acquisition, growth, transmission or clearance. Yet the extent to which SARS-CoV-2 is prone to within-host virus-virus and/or virus-bacteria interactions remains relatively unclear, may continue to evolve, and could have important consequences for epidemiological dynamics and clinical manifestations of antibiotic resistance.[[45]](https://sciwheel.com/work/citation?ids=13159055&pre=&suf=&sa=0&dbf=0)

**Page 6** Is death included here as a subset of discharge? Perhaps clarify one way or the other.

For simplicity, death is not considered explicitly in the model, although is accounted for implicitly as it factors into estimated lengths of stay for both standard and COVID-19 patients. This is now clarified in Figure 1 caption:

For simplicity, death is not explicitly considered.

**Page 9** Good to see the package being properly cited (and also in the supplementary material) but not in the caption to Figure 3 on page 13.

The citation has been added in Figure 3’s caption.

**Page 9** The model was populated using data from a long-term hospital but later the model is applied to other quite different settings. I think the validity of this needs to be discussed further. How typical is a long-term hospital of health care settings in general?

A great challenge of nosocomial research – whether *in silico* or *in vivo* – is the generalizability of findings. Healthcare facilities vary fundamentally in their population structures and underlying human behaviours (e.g. contact rates, procedures conducted, prescribing), so this French long-term care hospital, or any specific facility, could never be considered typical of all healthcare settings in general. In light of this, to ensure generalizability of pathogen transmission across settings (for both SARS-CoV-2 and MRB), we divided available transmission rates estimated from the literature by setting-specific contact rates, thus considering for each pathogen an intrinsic “transmission rate per contact” instead of a “transmission rate per unit time”, the latter inevitably varying greatly from hospital to hospital depending on its population and contact structures. This consideration is particularly important for our case study simulations, where we simulate pathogen transmission in settings with highly idiosyncratic contact behavior.

The contact standardization of transmission rates is described in Supplementary appendix section 1.3, but to make this information better available to the reader we have added the following sentence to the Methods (from line 273):

Pathogen transmission rates per unit of time estimated from the literature ($\beta$) were scaled to inter-individual contact rates per unit of time ($\kappa$) to approximate transmission rates per unit of contact ($\pi=\beta/\kappa$), facilitating generalizability across settings.

**Page 15, Figure 4b** I do not see how the diamond representing "all" can be positioned on the x{axis almost identically to the square for "policy" when there are clearly many points and the nabla for "caseload" well to the right of either. Is this due to excessive overprinting or have I misunderstood what the graph is supposed to be telling me?

This may be due to overprinting, resulting from a high number of data points (n=500) for each colour. Only a very small share of “all” points are significantly to the right of “policy” points. This is made clear below, where we provide Figure 4B but with its marginal distributions appended.

Although it may seem counterintuitive that the addition of caseload responses has little impact on the number of patient-days colonized, this reflects the non-additive nature of these diverse responses on epidemiological outcomes, and highlights insights that can be gained from mechanistic models into how overlapping mechanisms combine in non-obvious ways to impact infectious disease dynamics.

**Discussion** The authors motivate their work by pointing to conflicting results from empirical analysis of various datasets. Do their simulations go any way to resolving the different findings?

This is a good question. Given the idiosyncratic nature and limited generalizability of nosocomial outbreaks (as discussed above), it is unclear to what extent, if at all, conflicting results from empirical data obfuscate more general truths about impacts of COVID-19 on nosocomial resistance dynamics. We believe that a key advantage of our mechanistic modelling approach is that it helps to better understand the reasons why conflicting results from the literature may indeed be expected, due to great heterogeneity in how ecologically diverse bacterial species transmit across diverse healthcare settings and hence respond differently to pressures from COVID-19. This underlies why in our simulations different bacteria-facility pairs sometimes responded in opposite ways to the same COVID-19 response parameter(s). This motivates the main conclusion at the end of our discussion (from line 642):

In conclusion, this work has helped to disentangle how nosocomial SARS-CoV-2 outbreaks influence the epidemiological dynamics of multidrug-resistant bacteria. Our simulation-based approach facilitated the exploration of diverse scenarios and broad parameter spaces, helping to unravel the complexity and context-specificity of such impacts.

However, one key “generalizable” finding that emerged from our analysis is that surges in resistance mostly only occurred in settings with high underlying rates of bacterial transmission. We have added the following to the second paragraph of the discussion to better emphasize this (from line 534):

We further found that facilities with higher underlying rates of bacterial transmission were at greater risk of pandemic-associated surges in antibiotic resistance.

**Code** This is the best structured and documented set of code I have seen submitted as supplementary material.

We are very appreciative of this thoughtful comment.

I am not an expert in infectious disease control so I am unable to comment on whether the range of values which the authors have used to populate their model are plausible ones or whether the specific linkages are appropriate. It would be important to have a view from the clinical reviewers on this. Given the degree of detail here and the transparent code presented then anyone who subsequently disagrees with the authors' decisions can always re-run the model with new values.

We indeed hope that interested readers will consider using the code themselves as the reviewer suggests.

**Reviewer #2:** The paper reports the results of a modelling study aimed at estimating the impact of COVID-19 pandemic on mutidrug resistant bacteria transmission, in general and according to the type of bacteria, healthcare setting and timelinees and quality of response to COVID-19. The issue is relevant due to lack of conclusive data on the estimated impact of COVID-19 on antimicrobial resistance epidemiology. However, the strenght of the study results are highly dependant on the validity of assumptions made for building the study model. The model is based on parameters accounting for policy response and for caseload responses.

We thank the reviewer for the careful reading of our article and constructive comments. We agree that modeling results depend fundamentally on the assumptions made in building the model, and in particular on the parameter values underlying epidemiological processes. This was our motivation for conducting a range of sets of simulation accounting for variable levels of uncertainty in model parameters, in order to tease apart how variable COVID-19 responses impact a given hospital-bacterium pair (Figure 2), how fixed sets of COVID-19 responses impact a highly diverse sample of bacteria and healthcare facilities (Figures 3, 4), and how three scenarios of specific COVID-19 responses impact specific bacteria and healthcare facilities, using high-resolution contact data published in the literature (Figures 5, 6).

Two observations:
1. one of the parameters is "COVID prophylaxis", accounting for patient exposure to antibiotics for "prevention" of bacterial co-morbidities.
Antibiotic treatment is not given in the COVID population as prophylaxis, but as empirical therapy when a bacterial co-infection is suspected (because of x-ray or lab parameters suggestive of a co-existing bacterial pneumonia or in all critical patients given the diagnosis' challenge). Thus, the term prophylaxis is wrong…

We thank the reviewer for highlighting this and agree that antibiotics are not recommended for uncomplicated COVID-19 patients. However, high rates of antibiotic prescribing among COVID-19 patients during the first wave are well described in the literature, among whom only a minority have shown evidence of bacterial coinfection (meta-analyses of COVID-19 patients by Langford et al. [*Clin Microbiol Infect* 2021] and Rawson et al. [*Clin Infect Dis* 2020] estimated bacterial coinfection at 8.6% and 8%, and antibiotic therapy at 74.6% and 72%). Nonetheless, we acknowledge that the “COVID-19 prophylaxis” term in our model (which describes increased rates of antibiotic prescribing among COVID-19 patients for any reason) groups together instances of prescribing for observed or suspected bacterial coinfection, critical symptom presentation, or real instances of preventive or prophylactic use. We thus agree that prophylaxis is an inappropriate term for this parameter.

Throughout our paper, following the reviewer’s recommendation, we have now replaced all instances of “prophylaxis for COVID-19 patients/COVID-19 prophylaxis” with “antibiotic prescribing for COVID-19 patients/COVID-19 prescribing”.

…forecasting that all symptomatic patients receive antibiotics is a great overestimation of what should be the appropriate protocol for treament of these patients.

We absolutely agree that this would be an overestimate. Please note that in our main results we do not consider such high rates of antibiotic prescribing for COVID-19 patients. In the analysis of “generic bacteria” and “generic hospitals” we fix the COVID-19 prescribing parameter at an intermediate value ($\tau_{cp}=0.5$), which conservatively assumes excess antibiotic prescribing in only half of symptomatic COVID-19 patients. In the case study analysis, we then assume: (i) rates of COVID-19 prescribing equal to observed rates of bacterial coinfection in the “organized” scenario (approximately 5% to 15% of symptomatic COVID-19 patients) [Langford et al., *Clin Microbiol Infect* 2021], (ii) rates of prescribing equal to observed rates during the first wave for the “intermediate” response (68% to 80%) [Langford et al., *Clin Microbiol Infect* 2021], and finally (iii) very high rates of prescribing (90% to 100%) only in the “disorganized” response (Described in supplementary table S5).

2. Among the parameters used, the authors included the "abandoned stewardship" to take into account the increase in patients exposed to antibiotics due to reduction in antibiotic stewardship activities. A similar parameter has not been included for infection control and prevention. Hand hygiene is surely a core measure to prevent transmission but it is not the only one. Several studies have demonstrated an increase in the incidence of healthcare infections during the pandemic surges (eg the NHSN surveillance report). During the pandemic surge, infection control measures other than hand hygiene and DPI were not consistenlty applied. For example, healthcare workers did not have the time to change all the DPI (including isolation gowns, coveralls, protective suits) between patients and this has increased the likelihood of MRB transmission;  similarly, it was frequently reported a lower level of compliance with infection control measures when caring for invasive devices. The incapacity to mantain the infection control level previously reached in several hospitals have been reported as the driver of the observed increase in healthcare associated infections and, given that 2/3 of healthcare infections are due to MRB, the two phenomena are closely linked. The lack of accounting for a decreased capacity of infection control and prevention is an important study vulnus.

We certainly agree that surges in COVID-19 cases were accompanied in many instances by disorganization of infection control measures, for instance HCWs not having time to appropriately apply droplet precautions or doff/don/disinfect their PPE between visits with COVID-19 patients. In our model, we believe this phenomenon would be best captured by an increase in rates of high-risk patient-HCW contact during COVID-19 surges, translating to potential increases in patient-HCW transmission of MRB.

Such a parameter is in fact already included in our model. Our “cohorting disorganization” ($\tau_{cd}$) parameter was conceptualized as an increased rate of high-risk patient-HCW contact during COVID-19 surges (Table 1). When each of our COVID-19 response parameters were evaluated unilaterally, this disorganization parameter led to greater increases in MRB transmission than any other included parameter (Figure S2). In our analysis of generic MRB and generic hospitals, this parameter on its own also led to large increases in MRB incidence, in particular due to increases in transmission between patients and HCWs (Figure S12). Figure S13 perhaps best demonstrates how this parameter is most responsible for potential increases in both SARS-CoV-2 incidence and MRB incidence.

We believe that the reviewer has highlighted that our conceptualization and communication of this parameter was inadequate. Although the disorganization of cohorting may also increase any given HCW’s rate of contact with patients, this may have limited relevance for settings not having explicit cohorting structures in place at baseline, and is likely of secondary relevance relative to the disorganization of baseline infection control precautions. Based on this comment, we have thus reconceptualized this parameter as “care disorganization” and throughout our article we have replaced all instances of “cohorting disorganization” with “care disorganization”. For instance, in Table 1 care disorganization is now interpreted as:

Compromised ability of HCWs to adhere to IPC best practices (e.g. due to increased workload, PPE shortages) [Witt et al., *J Antimicrob Chemother* 2022]

It would be necessary to change the parameter "COVID prophylaxis" and to include a proxy accounting for the abandoned infection control program.

Both of these parameters have now been updated as per the reviewer’s recommendations.

The study shows that COVID-19 response lead to a reduction of MRB trasmission, while we know that, where data are available (NHSN), an increase of HAIs has been observed (see Baker et al. The Impact of COVID-19 on Healthcare-Associated Infections. Clin Infect Dis accepted paper).

To clarify, our primary findings are that surges in COVID-19 caseloads lead to increased MRB colonization incidence and higher resistance rates, while COVID-19 control measures lead to reduced MRB incidence with little impact on resistance rates. We now more carefully caution that our results should be interpreted as a theoretical disentangling of the complex mechanisms linking COVID-19 and AMR (from line 570):

In light of such complexity and data limitations, mathematical modelling is a powerful tool to help better understand and disentangle the complex, overlapping mechanisms linking COVID-19 and antibiotic resistance epidemiology. In this context, our model proposes theoretical explanations as to how outbreaks of an emerging pandemic pathogen like SARS-CoV-2 may be expected to exacerbate the spread of antibiotic resistance, and how the timely implementation of effective control measures can mitigate these impacts. Where data are lacking, mathematical modelling should continue to be exploited as a tool to understand mechanistic links between COVID-19 and antibiotic resistance beyond the early pandemic context explored here.

We certainly agree that many reports have highlighted increased HCAI, including the paper by Baker et al. already cited in our introduction (from line 143):

Yet in an analysis of microbiological data from 81 hospitals in the USA, infections due to MRSA, VRE and multidrug-resistant Gram-negative bacteria all spiked during local surges in COVID-19 cases.[[9]](https://sciwheel.com/work/citation?ids=11583261&pre=&suf=&sa=0&dbf=0)

Yet our work is focused on bacterial colonization and transmission dynamics, which are rarely observed and for which robust data (e.g. longitudinal asymptomatic carriage data) from during the pandemic are scarce. We have added the following sentence to our introduction to better emphasize this and motivate our study (from line 148):

These conflicting reports suggest that impacts of COVID-19 on antibiotic resistance likely depend on the particular population, setting and bacteria in question, and may be highly context-specific. Further, while several international studies have now reported on rates of healthcare-associated infection during the pandemic [Lastinger et al., *Infect Control Hosp Epidemiol* 2022; Rosenthal et al., *Int J Infect Dis* 2022], few have reported data on bacterial colonization or transmission, on rates of antibiotic resistance among colonized patients, nor on the putative mechanisms driving potential pandemic-related shits in antibiotic resistance epidemiology.[Witt et al., *J Antimicrob Chemother* 2022]

**Reviewer #3:** The submitted study is based on modelling showing the impact of COVID-19 and related containment actions on the spread of bacterial resistance in the hospitals. Although the evaluation has some limitations recognized by the authors, it provides interesting and useful insights on the effect of the control measures, taking into consideration the characteristics of the contexts and the way the measures are implemeted.

We thank the reviewer for their careful reading and constructive comments.

Considering the methodology used and the interest of the results in a public health perspective, in my opinion, the paper can be published with minor changes. The following suggestions are for the attention of the authors who can decide whether to take them into account or not:

- The ecological competition between resistant and antibiotic-susceptible bacteria is not always proven and in any case appears not to be complete. For example, according to some authors MRSA does not replace but goes (at least in part) in addition to MSSA (Boyce Effect). Infections/colonizations by susceptible and resistant bacteria can coexist or occur sequentially without specific solution of continuity. The assumption made could therefore constitute a limitation to be acknowledged.

We thank the reviewer for raising this point. To formalize a mechanism by which antibiotic use drives the spread of resistance, mathematical models assume that sensitive and resistant strains occupy the same ecological niche and thus compete within the host. Although such competition is borne out to a significant degree in experimental and clinical data, the reviewer correctly points out that co-colonization does occur and that real world dynamics are not nearly as simple as the “exclusive colonization” approach considered in our model. However, within-host strain competition dynamics remain poorly described and robust data are scarce. For this reason, most models, including ours, greatly simplify competition dynamics. For instance, in a review of population level antibiotic resistance models by Niewiadomska et al. [*BMC Med* 2019], only 8% of studies considered potential co-colonization/co-infection within the same host.

While introducing additional parameters to modulate strengths of intraspecific strain competition would surely influence some results quantitatively, we do not believe there would be a significant impact on our qualitative findings, in particular as all COVID-19 impacts evaluated aside from changes in antibiotic prescribing are not strain-specific. Nonetheless, we now acknowledge this limitation in our discussion (from line 635):

Bacterial strains are also conceptualized as competing exclusively for hosts, although co-colonization is widely observed *in vivo*. However, exclusive colonization is a common modelling approach for practical reasons (to keep models as simple as possible) and due to limited data describing within-host ecological competition dynamics [Niewiadomska et al., *BMC Med* 2019].

- It might be useful to describe in words in the body of the paper what is meant by "organized, intermediate or overwhelmed" responses.

The following text has been added to the results to better define response scenarios (from line 431):

Response scenarios were assumed to vary in terms of the COVID-19 policies put in place: for instance, in the organized response we assumed universal masking with N95 respirators, strict patient lockdown, a large increase in hand hygiene compliance, and COVID-19 prescribing only for patients experiencing bacterial coinfection; and in the disorganized response we assumed no masking, minimally effective patient lockdown, marginal improvement in hand hygiene and high rates of COVID-19 prescribing.

In the discussion, some considerations could also be introduced on how the availability of staff could influence the type of response. For example, the supplementary materials show how the "patient:HCW ratio" can influence compliance to the hand hygiene practice (Figure S1).

The following text has been added to the discussion to highlight the relevance of staffing in the possible response scenarios (from line 582):

It would also be helpful to evaluate impacts of variable availability of key resources, including masks, diagnostic tests, personal protective equipment and appropriately trained medical staff.

And the following has been elaborated upon to emphasize the importance of management systems and human resources in the efficiency of COVID-19 responses (from line 536):

In the context of poorly anticipated outbreaks of any novel respiratory pathogen, healthcare facilities that rapidly instate effective IPC measures and maintain stable staffing ratios while limiting unnecessary surges in antibiotic use may thus avoid concomitant surges in antibiotic resistance. However, putting such an organized response into action depends importantly on the financial and human resources available, and on the emergency management systems already in place.

- Some definitions are not immediately clear. For example, it could be useful to describe in words the meaning for the indicator "average delay between compliant handwashing events".

This definition has been clarified (Figure 2 caption):

The average delay between compliant HCW handwashing events ($\omega/\mathrm{day}^{-1}\times24 hours/day$), i.e. the average duration of transient bacterial carriage.

**Reviewer #4:** I am not legitimate to evaluate the mathematical model elaborated by the authors and the theoretical results it produced.
I put "minor revisions" because the PLOSMed website does not allow more precise choice such as "pending evaluation by other appropriate experts".
In addition, there is no window to fill with "reviewer comments to editor blind to authors", in which i may write that the assumptions underlying the model are disputable, as there is no "Simultaneous transmission dynamics of SARS-CoV-2 () and a commensal bacterium ()", and the transmission of drug-resistant bacteria occur mainly within the healthcare facilities, not outside of them.I have several remarks regarding the assumptions underlying this model, and regarding the interpretation of these results.

We thank the reviewer for the careful reading and relevant comments, which we have accounted for in this new version.

**Introduction:**
**- line 83:** there has not been such thing as an "antibiotic prophylaxis" in patients with Covid-19

We thank the reviewer for identifying this and agree with their interpretation. We have changed the term “COVID-19 prophylaxis” to “COVID-19 prescribing” throughout. Please also see our response to a similar comment from reviewer 2 on this topic.

**- line 85-86:** this hypothesis relies on the fact that drug-resistant bacteria are more transmitted out of the institutions (hospital, facility for the Elderly, etc), through daylife person-to-person contact (at home, at work, during leisures, during other interactions). Are the authors certain that it is the case in Europe? Such tranmission probably occur more frequently in healthcare institutions.

Here we refer specifically to interventions implemented within healthcare facilities and their impacts on antibiotic resistance dynamics within healthcare facilities. Community dynamics, while very important to the overall epidemiology of antibiotic resistance, are outside the scope of this study (as elaborated upon in the discussion). We have updated the wording to make this more clear (from line 133):

On the other, public health interventions implemented to control nosocomial SARS-CoV-2 transmission – including patient lockdown, hand hygiene education and provisioning of alcohol-based hand rub – may provide the unintended benefit of preventing bacterial transmission.

**Methods**
**- lines 119-120:** "Simultaneous transmission dynamics of SARS-CoV-2 () and a commensal bacterium ()": SARS-CoV-2 transmission occurs mainly by aerosol and droplets, meanwhile most of bacteria concerned by antibiotic resistance are transmitted by hand contact. Their transmission is not "simultaneous".

We believe this is a case of misinterpretation. By “simultaneous transmission” we meant that the model includes the transmission of both pathogens in the same population (a “co-infection model”), not that these pathogens are transmitted via the same processes. In the supplement we describe that SARS-CoV-2 is conceptualized as transmitting via aerosols/droplets (hence the important role for masking in limiting transmission) while bacteria are transmitted via contact and fomites (hence the important role for HCWs in facilitating spread among patients, and the key importance of hand hygiene for limiting bacterial transmission). For clarity, we have removed the word simultaneous and added the following text to the Methods (from line 174):

SARS-CoV-2 is conceptualized as transmitting via exhaled respiratory droplets/aerosols, while bacteria are conceptualized as transmitting via fomites and physical touch.

End of the abstract, and line 459 of the discussion, and line 555 in the conclusion: the authors cannot say that "Our model demonstrates how..." or "These findings thus highlight that...": being only in silico modelisation, it cannot "demonstrate" such things, but "suggest" or "bring arguments".

The wording in these sections has been nuanced as suggested.

**Discussion:**
**- lines 492-3:** the author speak of the impact of the pandemic "leading to reduced whole genome sequencing of bacterial isolates" as if it was something done daily by any hospital laboratory in France, which is not appropriate.

We did not mean to suggest that WGS is done daily on all isolates as this is clearly not the case. Rather, we meant that the pandemic has led to reduced WGS sequencing of bacterial isolates compared to pre-pandemic baseline. This is in part because, in many areas, existing laboratory infrastructure was shifted to sequence SARS-CoV-2 instead of bacterial isolates, as discussed in the CDC report that we cite alongside this text. We hope this proposed wording is now more clear (from line 561):

In many instances, SARS-CoV-2 testing and surveillance infrastructure has been repurposed from existing antimicrobial resistance infrastructure, leading to extensive gaps and delays in the reporting of antimicrobial resistance data since the onset of the pandemic, and a reduction in the number of bacterial isolates sent for whole genome sequencing.[[7]](https://sciwheel.com/work/citation?ids=12094546&pre=&suf=&sa=0&dbf=0)

**- line 505-6:** it is not possible to cite azithromycin and hydroxychloroquin as "treatment for COVID-19 and/or prophylaxis for bacterial coinfection" as they were not efficient, not recommended (appart from 2 weeks for HCQ in France in March 2020), and never demonstrated any effect on viral nor bacterial events in a Covid-19 context. It is not possible in the end of 2022 not to mention this when mentioning azithromycin and hydroxychloroquin.

We did not mean to suggest that hydroxychloroquine is effective COVID-19 treatment, nor recommended by guidelines. Nonetheless, azithromycin and hydroxychloroquine were frequently prescribed to COVID-19 patients early in the pandemic, as evidenced in the references cited. This is the context discussed here. We have updated this wording for clarity (from line 587):

An international surge in outpatient antibiotic consumption was observed initially in March 2020, associated primarily with antimicrobials frequently prescribed to COVID-19 patients early in the pandemic (e.g. azithromycin, hydroxychloroquine).[[31,32]](https://sciwheel.com/work/citation?ids=13338119,13338120&pre=&pre=&suf=&suf=&sa=0,0&dbf=0&dbf=0)
