# Supplementary material for: Collateral impacts of pandemic COVID-19 drive the nosocomial spread of antibiotic resistance: A modelling study
Source: PLoS Med. 2023 Jun 5;20(6):e1004240. doi: 10.1371/journal.pmed.1004240 (PMC10241372; doi:10.1371/journal.pmed.1004240)
Supplement: Attachment — Submitted filename: retour_aux_reviewers2.docx [file pmed.1004240.s004.docx]

We thank the editor and two reviewers for their time and feedback. Please find our replies below.

*INTRODUCTION
Please check for formatting errors pertaining to in-text reference callouts, for example line 122 “(e.g. Streptococcus pneumoniae).[3]” punctuation should follow parentheses as follows, “(e.g. Streptococcus pneumoniae) [3].” Similarly, line 137. Please check and amend throughout.*

These have been checked and amended throughout.

*Please indicate whether your study is novel and how you determined that.
If there has been a systematic review of the evidence related to your study (or you have conducted one), please refer to and reference that review and indicate whether it supports the need for your study.*

We have added two sentences in the introduction to provide context for the novelty of our work:

Mathematical modelling is a useful tool to help disentangle the mechanisms linking the transmission dynamics of co-occurring pathogens, especially when data are limited. However, recent work suggests that models describing impacts of COVID-19 on antibiotic resistance dynamics remain scarce [[15]](https://sciwheel.com/work/citation?ids=14791231&pre=&suf=&sa=0&dbf=0).

In particular, in the cited pre-print that is currently undergoing peer review, we conducted a systematic review of modelling studies indexed in PubMed that describe transmission dynamics of both SARS-CoV-2 and (antibiotic-resistant) bacteria. We found that, as of that search in August 2022, no transmission models had yet been published that include both SARS-CoV-2 and AMR, and in particular that describe impacts of the former on the latter. This supports our statement that models describing impacts of COVID-19 on antibiotic resistance dynamics remain scarce.

*FINANCIAL DISCLOSURE
Line 666 – please remove this statement from the main manuscript and include only in the relevant part of manuscript submission form when you re-submit your manuscript. It will be compiled as metadata.*

This statement has been removed.
